# A novel stroke mimic prediction score during in-hospital triage for suspected stroke patients: The Stroke Mimics Score (SMS)

**DOI:** 10.1177/23969873251338654

**Published:** 2025-05-15

**Authors:** Irene Scala, Marcello Covino, Pier Andrea Rizzo, Maurizio Bisegna, Davide Marchese, Simone Bellavia, Aldobrando Broccolini, Riccardo Di Iorio, Giacomo Della Marca, Valerio Brunetti, Francesco Franceschi, Mauro Monforte, Paolo Calabresi, Giovanni Frisullo

**Affiliations:** 1 Department of Neuroscience, Sense Organs, and Thorax, Fondazione Policlinico Universitario A. Gemelli IRCCS, Rome, Italy; 2 Catholic University of Sacred Heart, Rome, Italy; 3 Emergency Department, Fondazione Policlinico Universitario Agostino Gemelli IRCCS, Rome, Italy

**Keywords:** stroke, stroke mimics, prediction score, differential diagnosis

## Abstract

**Introduction:**

Early differential diagnosis between stroke mimics and cerebrovascular events is a major challenge in the Emergency Department (ED). The primary aim of this study was to identify diagnostic predictors of stroke mimics based on parameters acquired during the ED triage of patients with suspected stroke. Secondly, we aimed to develop a diagnostic score for early differential diagnosis. Moreover, we compared the diagnostic accuracy of our score with that of other two validated scores.

**Patients and methods:**

We included consecutive patients presenting to the ED of an urban teaching hospital for suspected stroke from 2015 to 2022 in the retrospective derivation cohort and during 2023 in the prospective validation cohort. Cerebrovascular events predictors were identified by logistic regression and were used to develop the Stroke Mimics Score (SMS). The diagnostic performance of SMS was assessed using the area under the receiver operating characteristics curves (AUROC) and the comparison with other diagnostic scores (FABS – Facial droop, Atrial fibrillation, Age, Systolic blood pressure, Seizure, Sensory symptoms– and TMS– TeleStroke Mimic score) was performed through DeLong method and Net Reclassification Index (NRI).

**Results:**

About 8648 patients were included in the study, 6998 in the retrospective cohort, and 1650 in the prospective cohort. In the retrospective cohort, 3266 (46.7%) patients had a final diagnosis of stroke mimic. Several variables collected by triage nurses independently predicted cerebrovascular event over stroke mimic diagnosis. The 10-variable SMS had excellent diagnostic performance in both the derivation and validation cohorts [AUROC 0.777 (95% CI: 0.766–0.788) and 0.774 (95% CI: 0.752–0.797), respectively] and outperformed FABS and TMS in all statistical comparisons.

**Discussion and conclusion:**

Several clinical variables elicited by triage nurses in the ED help to differentiate cerebrovascular events from stroke mimics in suspected stroke patients. The SMS is an easy-to-use score that could help selecting the best pathway for such patients.

## Graphical abstract

**Graphical abstract:**
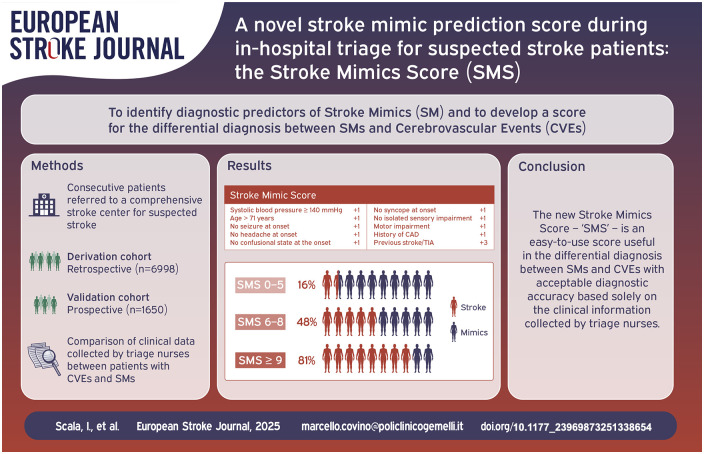

## Introduction

Ischemic stroke, a leading cause of death and disability worldwide,^1^ is a time-dependent disease that can benefit from revascularization procedures in the very early stages, already within the Emergency Department (ED).^2^ However, the principle of “time is brain” also firmly applies to brain hemorrhage, as timely diagnosis and treatment, mainly through bundle care protocols, are essential to prevent further neurological deterioration, minimize brain damage, and improve outcomes.^3^ Similarly, this principle holds to Transient Ischemic Attacks (TIAs), where prompt recognition allows physicians to implement all necessary measures to prevent ischemic stroke, which occurs in up to 21% of cases within the first 7 days after the transient episode.^4^

Since the time between the symptoms’ onset and the start of treatment is critical in determining patient outcomes, pre-hospital emergency personnel and physicians involved in the evaluation of patients with suspected stroke in the ED face several challenges in the timely recognition and management of stroke and TIAs. The fast and specific ability to detect patients with ischemic stroke who may benefit from a rapid and preferential diagnostic and treatment pathway, distinguishing them from patients with neurological symptoms due to non-cerebrovascular causes, remains the main challenge.^5^ Stroke mimics (SMs) are a group of medical conditions that present symptoms resembling those of a stroke but are caused by different underlying mechanisms, such as migraines, seizures, metabolic disturbances, and even psychological disorders. Accurately distinguishing between a real stroke and its imitations is crucial for several reasons. First, timely and appropriate treatment is crucial in the case of a stroke to reduce brain damage and improve the likelihood of functional recovery.^2^ Conversely, misdiagnosing an SM may lead to unnecessary administration of powerful clot-busting drugs, which pose risks to patients without offering therapeutic benefits.^6^ The proper care of the patient with suspected stroke begins in the pre-hospital phase, with the correct assignment of the stroke code by the paramedics of the emergency medical service (EMS) and the subsequent transport to the nearest stroke center with pre-notification to the referral ED.^7^

In patients admitted to the ED with symptoms suggestive of stroke, a significant proportion (5%–30%) may actually have SMs, such as migraine, seizures, brain tumors, infections, subdural hemorrhages, psychiatric disorders, and neuropathies.^8,9^ The over-triage and misdiagnosis impact significantly on the patient outcome, resource allocation and healthcare costs, especially in time-dependent conditions like stroke where clinical overlap of symptoms, time pressure, limited clinical experience, and lack of standardized protocols can increase the risk of errors, leading to inappropriate or delayed treatments.

In recent years, several clinical scales have been developed to distinguish strokes from mimics, some for use in pre-hospital settings,^10–12^ others designed for emergency physicians.^13–15^ These tools often require adequate and in-depth knowledge of the patient’s medical history, which is often difficult to achieve in the emergency context.

The primary aim of our study was to evaluate predictors of SM among clinical and physiological parameters collected by nursing staff during the ED triage in patients with suspected stroke. Secondarily, based on these predictors, we elaborated a diagnostic predictive score of stroke mimics able to distinguish between SM and acute CerebroVascular Events (CVEs). Finally, we compared the diagnostic performance of our score to those of the other two validated scores for the differential diagnosis between stroke and SM.

## Patients and methods

### Study design and population

In this single-center study conducted in a teaching urban hospital, we included all patients who presented to the ED with a suspected stroke diagnosis from 1 January, 2015, to 31 December, 2022, for retrospective enrollment (derivation cohort) and from 1 January, 2023, to 31 December, 2023, for prospective enrollment (validation cohort). Eligible patients were identified through automated data extraction from our hospital triage registry, selecting all patients categorized by triage nurses during the study period as “suspected stroke” from a drop-down menu containing various alternative diagnoses and who consequently were evaluated by stroke team neurologists. All those patients, following the request of ED physicians, were assessed by a stroke team consultant, a neurologist with expertise in cerebrovascular diseases.

The first part of the study was conducted on a retrospectively enrolled cohort of patients (derivation cohort). The variables collected for the derivation cohort were analyzed for univariate and multivariate correlation with the diagnosis of a CVE, defined as stroke (both ischemic and hemorrhagic) and Transient Ischemic Attack (TIA) versus diagnosis of SM at hospital discharge to identify early predictors of stroke. Independent predictors of CVEs were used to develop the Stroke Mimics Score (SMS), a tool designed to assist physicians in early differential diagnosis between these two conditions. In the second part of the study, we validated the SMS on a prospective cohort of patients (validation cohort) admitted to our’s hospital ED during the following year for suspected stroke. A detailed list of inclusion and exclusion criteria and setting of pre-hospital and hospital stroke pathways are available in the Supplemental Materials.

The study was conducted in accordance with the Strengthening the Reporting of Observational Studies in Epidemiology (STROBE) guidelines for observational studies.

### Study variables

The data analyzed in this study were automatically extracted from our hospital’s triage registry, where triage nurses prospectively record information on all patients admitted to the ED. Data on in-hospital mortality and the discharge diagnoses of included patients were collected through automated extraction from the electronic hospital registry, which is prospectively updated by our hospital’s physicians. Details on the collected variables and the process of triage registry completion are available in the Supplemental Materials.

The main discharge diagnosis was categorized into one of three possible options: stroke, SM, or TIA. For specific diagnostic definitions, refer to the Supplemental Materials. We grouped all patients with a primary discharge diagnosis of a CVE, defined as ischemic stroke, TIA, and cerebral hemorrhage into one group and compared them with those diagnosed with SM.

Differential diagnosis between CVEs and SMs was based on medical history, serial neurological examinations, and at least one neuro-radiological assessment (brain CT or angiography-CT and/or MRI). Furthermore, patients underwent several additional clinical investigations, including a cardiological workup (echocardiogram and electrocardiographic monitoring), electroencephalography, blood tests, and carotid duplex ultrasound if deemed necessary by the treating physicians. The final diagnosis was based on the consensus of the stroke team neurologists. In cases of diagnostic uncertainty, the most likely diagnosis was used.^16^

Finally, to compare the SMS with other validated scores for the discrimination of CVEs and SMs, we calculated the “absence of Facial droop, negative history of Atrial fibrillation, Age <50 years, systolic Blood pressure <150 mm Hg at presentation, history of Seizures, and isolated Sensory symptoms without weakness at presentation” (FABS) score^14^ and the TeleStroke Mimic score (TMS)^11^ for patients in the derivation and validation cohorts. The FABS score is a six-item score designed for stroke neurologists to facilitate the rapid identification of SMs among patients presenting with suspected stroke and negative brain CT scan in the ED setting. Since higher FABS scores are associated with a higher probability of SM diagnosis, whereas SMS follows an opposite trend, we used the inverted FABS score in statistical comparisons (i.e. higher scores indicate a higher risk of CVE diagnosis at discharge). Conversely, the TMS score is a six-item score intended to be completed with information gathered during a teleconsultation with the stroke neurologists from a comprehensive stroke center for patients with suspected stroke admitted to the ED of a spoke hospital and it follows the same trend as SMS (higher scores indicate a higher risk of CVE diagnosis at discharge). Both scores consist of clinical parameters that can be easily collected during the emergency evaluation of stroke patients and are intended to be completed by medical personnel. A table listing the individual items of the FABS and the TMS scores is available in the Supplemental Materials (Table S1). We selected these two scores as comparators for our score to assess its diagnostic performance against tools requiring completion by medical personnel, in order to verify the accuracy and reliability of the triage nursing staff evaluation in the differential diagnosis between CVE and SM.

### Statistical analysis

Qualitative variables are expressed as absolute and relative percentage frequencies. The Gaussian distribution of the study variables was assessed using the Shapiro-Wilk test. Quantitative variables are reported as median and interquartile range (IQR). Comparisons among groups were performed using the χ2-test or Fisher’s exact test for qualitative variables and the Mann-Whitney *U*-test or the Kruskal-Wallis test for numerical variables, as appropriate. Missing data were not imputed.

A stepwise logistic regression model was performed to identify predictors of CVE diagnosis, including variables collected during the triage phase of patients with suspected stroke that obtained a *p* < 0.100 (suggestive predictors) in the univariate comparison between the two study groups (e.g. SM vs CVE). To avoid logistic model instability, we used both cluster analysis and the adjusted weights in linear regression analysis to exclude significant variables predicting <5% of events. The final logistic model included 10 variables with a strong independent association with CVE or SM diagnosis in the retrospective cohort. The goodness of fit of the logistic regression model was assessed by the Hosmer–Lemeshow test. To improve model fitting, and make more straightforward the score calculation, we dichotomized the continuous variables by using the Youden Index J of a Receiver Operating Characteristic (ROC) analysis.

Once the factors to include in the score were identified, we assigned a value to each variable by creating a simple linear regression model including all the identified predictors. The coefficients of these predictors represent the predicted change in CVE diagnosis imparted by each variable by itself, whereas the adjusted coefficients indicate the relative contribution of each factor in the model. Based on these premises, we assigned different points to each variable using the standardized coefficients as a guide for the relative contribution of each item. After calculating the score, we assessed its diagnostic performance on a cohort of patients prospectively enrolled by calculating the area under the ROC curves (AUROC).

We then compared the performance of the SMS with the FABS and TMS scores. The discrimination (i.e. the accuracy in discriminating two discharge diagnoses) of the three scores was again measured through the AUROC. To identify the score with the highest accuracy, we compared the AUROCs of the scores using the DeLong et al.^17^ method. The overall performance improvement of stroke risk prediction of SMS versus FABS and TMS was also evaluated through the Net Reclassification Index (NRI).^18^

The calibration of the score (i.e. the ideally linear relationship between the increase in the score and the probability of a CVE diagnosis vs SM or vice versa) was visually evaluated by plotting the number of events for each score value. To enhance the clinical applicability of the final score, we compared the observed (confirmed CVE diagnosis) versus expected events (suspected stroke diagnosis) at each score level and grouped similar values into three risk groups, obtaining the SMS group score (SMSg). The AUROC of the SMSg was then compared by the DeLong et al.^17^ method to the AUROC of the simplified version of the other two scores (i.e. FABS and TMS) to ascertain significant differences. Since the FABS score has an opposite trend (i.e. higher scores are suggestive of a higher probability of an SM diagnosis), we inverted the direction of the ROC curve to compare it to the other scores.

Statistical significance was set at a two-tailed *p*< 0.05. Statistical analyses were performed through SPSS v26^®^ (IBM, Armonk, NY, USA) and MedCalc Statistical Software version 18 (MedCalc Software Ltd, Ostend, Belgium; https://www.medcalc.org; 2023).

## Results

Of the 9694 patients referred to our ED for suspected stroke from 2015 to 2022, 6998 met the study criteria and were included in the retrospective derivation cohort (a detailed flowchart of the enrollment process is available in Figure S1). During the following year, 1650 patients met the criteria to be enrolled in the prospective cohort for score validation. Details on the characteristics of the entire study population and the comparison between the two cohorts of patients are available in Table S2. In the retrospective cohort, 3266 (46.7%) patients had a discharge diagnosis of SM. Among the 3732 (53.3%) patients with a discharge diagnosis of a CVE, 472 (12.6%) had a cerebral hemorrhage, 2921 (78.2%) had an ischemic stroke, and 339 (9.1%) had a TIA.

**Figure 1.**
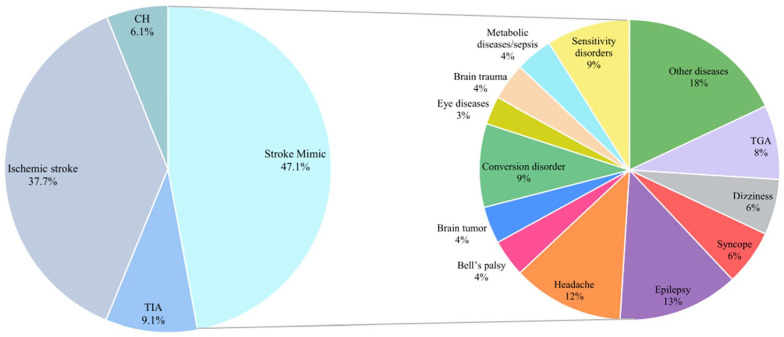
Discharge diagnoses of patients in the derivation cohort. TIA: transient ischemic attack; CH: cerebral hemorrhage; TGA: transient global amnesia.

At the end of the diagnostic process, most patients with SM received a diagnosis of epilepsy (13%), followed by headache (12%, mostly migraine with aura [74.4%]), conversion disorder (9%), sensory disorders (9%), and transient global amnesia (8%; Figure 1).

The median age of patients included in the retrospective cohort was 74.0 (61.0–83.0) years and there was a slight predominance of male patients (51.1%). Median NIHSS score at admission was 8.0 (3.0–16.0) and language (52.1%) and motor disorders (58.1%) were the most frequent symptoms at admission. Among comorbidities, hypertension (76.0%), a history of previous stroke and/or TIA (36.2%), coronary artery disease (26.5%), and kidney failure (24.6%) were the most frequently represented. Almost 11% of patients underwent revascularization treatments for acute stroke, 69.9% were hospitalized, and 8.6% died during hospitalization.

Several differences emerged between patients with a discharge diagnosis of CVE and those with SM.

Patients with a discharge diagnosis of a CVE arrived at the ED more frequently via emergency medical service, whereas patients with SMs tended to arrive as walk-ins (*p* < 0.001). Furthermore, patients in the CVE group had shorter median onset-to-door times than SM patients (*p* < 0.001).

Patients with CVEs were significantly older than the others (*p* < 0.001). Regarding the physiological parameters collected at the time of ED access, stroke patients had higher median systolic and diastolic blood pressure (respectively, *p* < 0.001 and *p* = 0.005), but lower heart rates (*p* < 0.001) than SM patients. Even if the median NIHSS score was similar, all the neurological symptoms at admission differed between the two groups (all *p* < 0.001). In particular, a sensory deficit, either isolated or not, syncope, seizures, dizziness, headache, and confusional state at onset were more frequently observed in patients with a discharge diagnosis of SM, whereas motor and language disorders, facial droop, and confusional state at onset were more common in those with CVEs. A graphical representation of the onset symptoms in patients from both the derivation and validation cohorts is available in Figure S2. Patients with CVEs had a higher comorbidity burden (*p* < 0.001), mostly explained by the higher prevalence of diabetes, hypertension, congestive heart failure, clinical history of coronary artery disease, and previous stroke/TIA (all *p* < 0.001). On the other hand, kidney failure (*p* < 0.001) was more frequent in patients of the SM group. The detailed results of the above-mentioned comparisons are presented in Table 1.

**Table 1.** The univariate comparison between patients of the derivation cohort with a discharge diagnosis of CVE and those with a diagnosis of SM.

Baseline variables		Derivation cohort (*n* = 6998)	CVEs (*n* = 3732)	Stroke mimics (n = 3266)	Unadjusted *p*-value
Demographics	Age (years)	74.0 (61.0–83.0)	77.0 (66.0–84.0)	69.0 (54.0–80.0)	**<0.001**
	Sex (female)	3420 (48.9%)	1789 (47.9%)	1631 (49.9%)	*0.095*
Triage	Emergency	2802 (40.0%)	1959 (52.5%)	843 (25.8%)	**<0.001**
	Urgency	3195 (45.7%)	1441 (38.6%)	1754 (53.7%)	
	Minor urgency	1001 (14.3%)	332 (8.9%)	669 (20.5%)	
Mode of ED arrival	Emergency medical service	3678 (52.6%)	2192 (58.7%)	1486 (45.5%)	**<0.001**
Onset to door time (h)	<3	3548 (50.7%)	1974 (52.9%)	1574 (48.2%)	**<0.001**
	3–6	1258 (18.0%)	694 (18.6%)	564 (17.3%)	
	6–12	642 (9.2%)	325 (8.7%)	317 (9.7%)	
	12–24	1550 (22.2%)	739 (19.8%)	811 (24.8%)	
Vitals (ED admission)	Heart rate (bpm)	81.0 (71.0–92.0)	80.0 (70.0–90.0)	83.0 (73.0–94.0)	**<0.001**
	Systolic blood pressure (mmHg)	152.5 (135.0–170.0)	156.0 (140.0–174.0)	150.0 (130.0–167.0)	**<0.001**
	Diastolic blood pressure (mmHg)	86.0 (77.0–97.0)	87.0 (78.0–99.0)	85.0 (76.0–95.0)	**0.005**
	SaO_2_ (%)	97.0 (96.0–98.0)	97.0 (96.0–98.0)	97.0 (96.0–98.0)	**<0.001**
Neurological symptoms (ED admission)	NIHSS	8.0 (3.0–16.0)	7.0 (3.0–16.0)	8.0 (3.0–16.0)	0.725
	Altered consciousness	917 (13.1%)	609 (16.3%)	308 (9.4%)	**<0.001**
	Confusional state	1409 (20.1%)	619 (16.6%)	790 (24.2%)	**<0.001**
	Language disorder	3645 (52.1%)	2256 (60.5%)	1389 (42.5%)	**<0.001**
	Motor impairment	4068 (58.1%)	2429 (65.1%)	1639 (50.2%)	**<0.001**
	Sensory impairment	852 (12.2%)	280 (7.5%)	572 (17.5%)	**<0.001**
	Isolated sensory impairment	161 (2.3%)	27 (0.7%)	134 (4.1%)	**<0.001**
	Facial drop	933 (13.3%)	552 (14.8%)	381 (11.7%)	**<0.001**
	Headache	898 (12.8%)	294 (7.9%)	604 (18.5%)	**<0.001**
	Dizziness	554 (7.9%)	160 (4.3%)	394 (12.1%)	**<0.001**
	Seizure	448 (6.4%)	134 (3.6%)	314 (9.6%)	**<0.001**
	Syncope	682 (9.7%)	258 (6.9%)	424 (13.0%)	**<0.001**
Comorbidities	Charlson comorbidity index	3.0 (1.0–5.0)	4.0 (2.0–6.0)	2.0 (1.0–4.0)	**<0.001**
	History of CAD	1853 (26.5%)	1020 (27.3%)	833 (25.5%)	*0.084*
	Hypertension	4827 (76.0%)	2852 (81.3%)	1975 (69.3%)	**<0.001**
	Atrial fibrillation	834 (11.9%)	439 (11.8%)	395 (12.1%)	0.670
	Congestive heart failure	2031 (29.0%)	938 (25.1%)	1093 (33.5%)	**<0.001**
	Peripheral artery disease	1113 (15.9%)	783 (21.0%)	330 (10.1%)	**<0.001**
	Previous TIA/stroke	2536 (36.2%)	2033 (54.5%)	503 (15.4%)	**<0.001**
	Major neurocognitive disorder	338 (4.8%)	163 (4.4%)	175 (5.4%)	*0.054*
	COPD	228 (3.3%)	124 (3.3%)	104 (3.2%)	0.745
	Liver disease	82 (1.2%)	39 (1.0%)	43 (1.3%)	0.292
	Diabetes	1101 (15.7%)	686 (18.4%)	415 (12.7%)	**<0.001**
	Kidney failure	1723 (24.6%)	747 (20.0%)	976 (29.9%)	**<0.001**
	Active cancer	421 (6.0%)	228 (6.1%)	193 (5.9%)	0.726

Significant differences are indicated in bold, while suggestive differences are shown in italics. CVEs: cerebrovascular events; ED: emergency department; SaO_2_: peripheral oxygen saturation; NIHSS: National Institute of Stroke Scale; CAD: coronary artery disease; TIA: transient ischemic attack; COPD: chronic obstructive pulmonary disease.

Nearly 20% of patients in the CVE group underwent revascularization treatments, in contrast with 1% in the SM cohort (*p* < 0.001). Patients with CVEs were more frequently hospitalized (*p* < 0.001), especially in the neurology department (*p* < 0.001) and had significantly longer median length of hospitalization than those with SMs (*p* < 0.001). Due to the higher hospitalization rates, 2739 CVE patients (73.4%) underwent brain MRI in the acute or subacute phase compared to 1389 patients (42.5%) in the SM group. Finally, patients with a discharge diagnosis of CVE had a significantly higher in-hospital mortality rate than those with SM (*p* < 0.001). Please refer to Table S3.

Table S4 presents the results of the univariate comparisons between patients in the validation cohort with a discharge diagnosis of SM and those with a diagnosis of CVE.

### Factors associated with stroke diagnosis in the retrospective cohort

The multivariable logistic regression identified independent predictors of CVE among parameters collected during the triage phase (Table 2). A history of previous stroke/TIA was the strongest predictor of CVE diagnosis, with an Odds Ratio (OR) of 6.237 (*p* < 0.001), followed by the absence of seizures (OR 3.846, *p* < 0.001), syncope (OR 2.124, *p* < 0.001), or isolated sensory disorder (OR 2.243, *p* < 0.001) at symptom onset. Other factors independently associated with CVE diagnosis at discharge were age ⩾ 71 years, systolic blood pressure at the time of ED arrival ⩾140 mmHg, absence of confusional state and headache at symptoms’ onset, clinical history of CAD, and a motor deficit at the neurological examination.

**Table 2.** Multivariable logistic regression to find independent predictors of CVE in the derivation cohort.

Covariates	OR (95% CI)	*p*-Value
Age > 71 years	1.764 (1.573–1.979)	<0.001
SBP ⩾ 140 mmHg at triage assessment	1.296 (1.160–1.447)	<0.001
No seizure at onset	3.846 (3.037–4.870)	<0.001
No confusional state at onset	1.905 (1.663–2.184)	<0.001
No syncope at onset	2.124 (1.765–2.557)	<0.001
No isolated sensory disorders	2.243 (1.884–2.671)	<0.001
Motor disorders at onset	1.488 (1.333–1.662)	<0.001
No headache at onset	1.828 (1.543–2.165)	<0.001
Previous stroke/TIA	6.237 (5.512–7.056)	<0.001
History of CAD	1.348 (1.193–1.523)	<0.001

CVE: cerebrovascular event; OR: odds ratio; CI: confidence interval; SBP: systolic blood pressure; TIA: transient ischemic attack; CAD: coronary artery disease.

### Score development on the retrospective cohort

Based on independent predictors of CVE diagnosis identified through the multivariable logistic regression, we developed the SMS by assigning points to each of the identified variables. The point values were determined using the β-coefficients obtained by each variable in an adjusted linear regression model (Table S5). The final SMS is presented in Table 3.

**Table 3.** The Stroke Mimics Score (SMS). Higher scores suggest a higher probability of CVE diagnosis.

Score items	Score points
Systolic blood pressure ⩾ 140 mmHg	+1
Age > 71 years	+1
No seizure at onset	+1
No headache at onset	+1
No confusional state at the onset	+1
No syncope at onset	+1
No isolated sensory impairment	+1
Motor impairment	+1
History of CAD	+1
Previous stroke/TIA	+3

CAD: coronary artery disease; TIA: TRANSIENT ISCHEMIC ATTACK.

**Figure 2.**
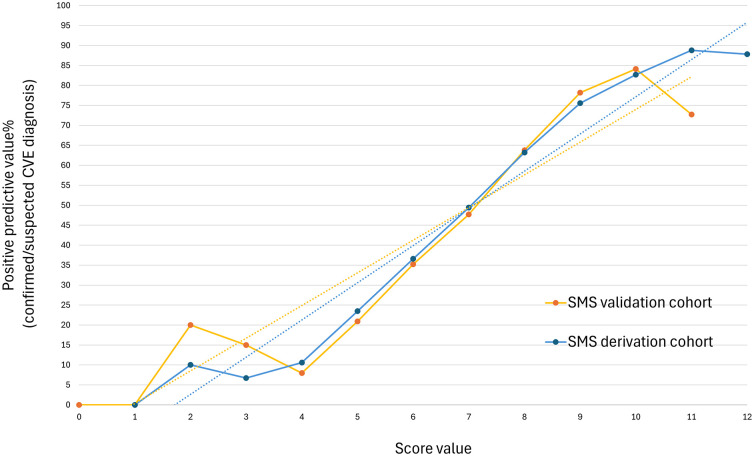
Calibration curves of the SMS in the derivation and validation cohorts. CVE: cerebrovascular events; SMS: Stroke Mimics Score.

Overall, 1377 (19.1%) patients had a SMS between 0 and 5 and were considered at low-risk for CVEs (<25% probability), the 3349 (47.9%) patients who obtained a SMS between 6 and 8 had a probability comprised between 25% and 75% of a discharge diagnosis of a CVE and were considered at moderate-risk, while 2272 (26.5%) individuals had a SMS ⩾ 9 and were considered at high-risk for CVEs (>75% probability). The calibration curves of the SMS for both the derivation and the validation cohorts of patients are presented in Figure 2. A visual representation of the SMS calibration, along with the classification into risk categories and a comparison with the FABS and TMS scores, is available in Table S6 and Figure S3. The diagnostic performance of the three scores in patients of the validation cohort is available in Table S7 and Figure S4.

### Score performance and comparison with FABS and TMS scores

The median SMS value in patients with a discharge diagnosis of CVEs was significantly higher than in those with a discharge diagnosis of SMs [8.0 (7.0–10.0) vs 6.0 (5.0–7.0); *p* < 0.001], as was the TMS score [15.0 (13.0–17.0) vs 12.0 (10.0–15.0); *p* < 0.001), whereas the FABS score showed an opposite trend [2.0 (2.0–3.0) vs 3.0 (2.0–3.0); *p* < 0.001] in the derivation cohort. Similar values were found in the validation cohort (please refer to Table S4).

**Figure 3.**
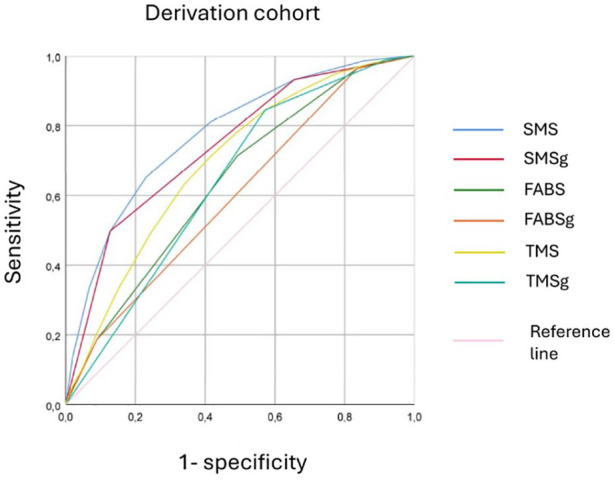
Receiver Operating Characteristic curves of the SMS, FABS, and TMS scores, in both their continuous and grouped versions in the derivation cohort. SMS: Stroke Mimic Score; SMSg: Stroke Mimic Score grouped; FABSg: FABS grouped; TMS: Telestroke Mimic Score; TMSg: Telestroke Mimic Score grouped.

The SMS had acceptable diagnostic accuracy for patients in the derivation cohort, obtaining an AUROC of 0.777 (95% CI: 0.766–0.788) for its continuous version and an AUROC of 0.742 (95% CI: 0.731–0.754) for its grouped version (both *p* < 0.001). The FABS and the TMS had lower accuracies than the SMS in both the continuous [AUROC 0.639 (95% CI: 0.626–0.652) and AUROC 0.694 (95% CI: 0.681–0.706), respectively; both *p* < 0.001] and the grouped versions of the scores [AUROC 0.599 (95% CI: 0.586–0.612) and AUROC 0.641 (95% CI: 0.627–0.654), respectively; both *p* < 0.001]. Please refer to Figure 3.

In the prospective cohort, the SMS obtained similar results, with an AUROC of 0.774 (95% CI: 0.752–0.797) for the continuous version and an AUROC of 0.744 (95% CI: 0.721–0.768) for the SMSg (both *p* < 0.001). Again, both FABS and the TMS scores performed worse than SMS (Figure S5) in the validation cohort. Details on the statistical comparisons among the ROC curves and the extended results for the validation cohort can be found in Tables S8 and S9.

Finally, we compared the diagnostic accuracy of SMSg with those of the grouped version of FABS and TMS using the NRI. The NRI confirmed that the SMSg had a better accuracy than both FABSg [NRI 0.435 (0.406–0.454); *p* < 0.001)] and TMSg [NRI 0.254 (0.222–0.286); *p* < 0.001)].

## Discussion and conclusion

The differentiation between acute CVEs and SM represents an intricate challenge that, especially in the early moments after symptoms onset, can lead to delays or misdirection of the in-hospital pathway of patients with acute stroke. The correct classification of patients with suspected stroke in the triage phase represents the critical element since it is based on few and fragmentary information collected by the nursing staff. To address this issue, we identified clinical and physiological parameters collected by the nursing staff during the triage phase of patients admitted to the ED for suspected stroke able to early distinguish CVEs from SMs. Based on these independent predictors, we developed a quick, easy-to-use diagnostic tool, the SMS, designed to assist ED physicians and stroke neurologists in the diagnostic process. Finally, we found that the diagnostic performance of our score was better than that of previously published scores such as TMS and FABS.

In our retrospective cohort of 6998 patients, SMs accounted for 46.7% of the final discharge diagnosis. This finding aligns with previous literature, in which the prevalence of stroke-mimicking conditions in the ED ranges from just over 20% up to 65%.^11,13,15,19^

In line with previous studies, in our cohort of patients, CVE patients were older,^11,14,16^ with a higher burden of cardiovascular risk factors,^11,13,14,16^ and higher median values of systolic and/or diastolic blood pressure at the time of the ED presentation^11,14^ compared to SM patients. On the other hand, we found no significant sex difference in the prevalence of SM, a result that aligns with most studies,^11,13,15,16,20^ but contrasts with others.^14,21^ Neurological manifestations at onset differed remarkably between patients with SMs and CVEs. Seizure, sensory deficits (especially if isolated), dizziness, headache, loss of consciousness, and confusional state were more frequent in SM patients, whereas motor impairment, language disorders, and facial droop in CVE patients, similar to what previously reported.^22^

Considering the above-mentioned differences in comorbidities and clinical presentation, collected during the triage phase at ED admission by the nursing staff, we developed a diagnostic score, the SMS, capable of early discrimination between CVEs and SMs with fair diagnostic accuracy.

To further facilitate the decision-making process, we also developed a grouped version of the score, the SMSg, which allows for the classification of patients into three risk categories: low risk (<25% probability of CVE), high risk (>75% probability of CVE), and intermediate risk. This stratification could be useful for prioritizing high-risk patients’ pathways, through the pre-alerting of the stroke team, neuroradiologists, laboratory personnel, etc., and for identifying a low-risk category for CVE for whom a clinical assessment by the emergency physician before the initiation of the stroke pathway could avoid unnecessary expenditure of time and resources. Furthermore, although the SMS was tested in the hospital triage phase, its user-friendly nature based on data collected from non-medical and/or paramedical personnel, makes it easily adaptabe to the pre-hospital phase, including use by EMS nurses and paramedics. After appropriate risk stratification using the different SMSg risk categories, subjects with a high CVE risk could be directly referred to primary stroke centers. Conversely, those with a low CVE risk could be transferred to local non-comprehensive stroke centers, relieving the pressure of emergency departments of comprehensive stroke centers.

An early differential diagnosis between CVEs and SMs is essential to avoid unnecessary diagnostic procedures, specialist consultations, and thrombolytic treatments, which in our study was carried out in 1% of SM patients, exposing them to unwarranted adverse events^6,23^ and increasing the burden on health economic budget.^24^ Furthermore, an early and accurate diagnosis ensures that a brain hemorrhage is not overlooked, allowing for the appropriate treatment to be given, reducing the risk of additional brain tissue damage and cerebral edema, prompt surgical intervention, and the quick administration of antidotes or medications that can reverse the effects and stop the bleeding in patients on oral anticoagulant treatment.^3^ Furthermore, an early differential diagnosis could help speed up the management of patients with acute CVE, reducing the overload of hospital resources by patients with SMs.

Several scores have already been developed for the differential diagnosis between CVEs and SMs,^10–15^ some of them are based on assessments performed by medical staff during an on-site evaluation in the ED,^14,15^ a teleconsultation,^11^ or during the patient’s in-hospital stay,^13^ and others on parameters collected by the paramedical staff during the pre-hospital phase.^10,12^ In this regard, our score is based on information collected by the nursing staff during the ED triage phase and could be calculated before a medical visit.

We then compared our score to two other validated scores: a score developed on variables collected during teleconsultation with a stroke neurologist (TMS),^11^ and a score based on the stroke neurologist assessment in the ED (FABS).^14^ Unlike the SMS, both scores were developed on highly selected patient populations, who have already undergone several diagnostic examinations, often including a brain CT scan, and the information used for score development was collected by stroke neurologists. Moreover, in our study, both FABS and TMS had lower diagnostic accuracy than SMS.

Our study has several limitations: The single-center design did not allow us to test the SMS on different populations of patients with suspected stroke.^25^ Secondly, we must consider the retrospective development of the score, which did not allow us to collect all the variables necessary for comparison with other validated scores.^15^ Thirdly, given that about 30% of patient were not hospitalized, that less than 50% were hospitalized in the neurology department for work-up, and that and MRI was not obtained in about 40% of patients, the classification as CVE and SM based on the discharge diagnosis was potentially incorrect in some patients. Finally, our score is not able to properly address the issue of CVE underdiagnosis, namely stroke chameleons, in the ED.

To our knowledge, this is the first study developing a score for the differential diagnosis between SMs and CVEs with acceptable diagnostic accuracy based solely on the clinical information collected by triage nurses. However, future multicenter prospective studies should be conducted in heterogeneous patient populations and in a wider variety of clinical settings.

## Supplemental Material

sj-docx-1-eso-10.1177_23969873251338654 – Supplemental material for A novel stroke mimic prediction score during in-hospital triage for suspected stroke patients: The Stroke Mimics Score (SMS) Supplemental material, sj-docx-1-eso-10.1177_23969873251338654 for A novel stroke mimic prediction score during in-hospital triage for suspected stroke patients: The Stroke Mimics Score (SMS) by Irene Scala, Marcello Covino, Pier Andrea Rizzo, Maurizio Bisegna, Davide Marchese, Simone Bellavia, Aldobrando Broccolini, Riccardo Di Iorio, Giacomo Della Marca, Valerio Brunetti, Francesco Franceschi, Mauro Monforte, Paolo Calabresi and Giovanni Frisullo in European Stroke Journal

sj-docx-2-eso-10.1177_23969873251338654 – Supplemental material for A novel stroke mimic prediction score during in-hospital triage for suspected stroke patients: The Stroke Mimics Score (SMS) Supplemental material, sj-docx-2-eso-10.1177_23969873251338654 for A novel stroke mimic prediction score during in-hospital triage for suspected stroke patients: The Stroke Mimics Score (SMS) by Irene Scala, Marcello Covino, Pier Andrea Rizzo, Maurizio Bisegna, Davide Marchese, Simone Bellavia, Aldobrando Broccolini, Riccardo Di Iorio, Giacomo Della Marca, Valerio Brunetti, Francesco Franceschi, Mauro Monforte, Paolo Calabresi and Giovanni Frisullo in European Stroke Journal

sj-docx-3-eso-10.1177_23969873251338654 – Supplemental material for A novel stroke mimic prediction score during in-hospital triage for suspected stroke patients: The Stroke Mimics Score (SMS) Supplemental material, sj-docx-3-eso-10.1177_23969873251338654 for A novel stroke mimic prediction score during in-hospital triage for suspected stroke patients: The Stroke Mimics Score (SMS) by Irene Scala, Marcello Covino, Pier Andrea Rizzo, Maurizio Bisegna, Davide Marchese, Simone Bellavia, Aldobrando Broccolini, Riccardo Di Iorio, Giacomo Della Marca, Valerio Brunetti, Francesco Franceschi, Mauro Monforte, Paolo Calabresi and Giovanni Frisullo in European Stroke Journal

sj-docx-4-eso-10.1177_23969873251338654 – Supplemental material for A novel stroke mimic prediction score during in-hospital triage for suspected stroke patients: The Stroke Mimics Score (SMS) Supplemental material, sj-docx-4-eso-10.1177_23969873251338654 for A novel stroke mimic prediction score during in-hospital triage for suspected stroke patients: The Stroke Mimics Score (SMS) by Irene Scala, Marcello Covino, Pier Andrea Rizzo, Maurizio Bisegna, Davide Marchese, Simone Bellavia, Aldobrando Broccolini, Riccardo Di Iorio, Giacomo Della Marca, Valerio Brunetti, Francesco Franceschi, Mauro Monforte, Paolo Calabresi and Giovanni Frisullo in European Stroke Journal

sj-docx-5-eso-10.1177_23969873251338654 – Supplemental material for A novel stroke mimic prediction score during in-hospital triage for suspected stroke patients: The Stroke Mimics Score (SMS) Supplemental material, sj-docx-5-eso-10.1177_23969873251338654 for A novel stroke mimic prediction score during in-hospital triage for suspected stroke patients: The Stroke Mimics Score (SMS) by Irene Scala, Marcello Covino, Pier Andrea Rizzo, Maurizio Bisegna, Davide Marchese, Simone Bellavia, Aldobrando Broccolini, Riccardo Di Iorio, Giacomo Della Marca, Valerio Brunetti, Francesco Franceschi, Mauro Monforte, Paolo Calabresi and Giovanni Frisullo in European Stroke Journal

sj-docx-6-eso-10.1177_23969873251338654 – Supplemental material for A novel stroke mimic prediction score during in-hospital triage for suspected stroke patients: The Stroke Mimics Score (SMS) Supplemental material, sj-docx-6-eso-10.1177_23969873251338654 for A novel stroke mimic prediction score during in-hospital triage for suspected stroke patients: The Stroke Mimics Score (SMS) by Irene Scala, Marcello Covino, Pier Andrea Rizzo, Maurizio Bisegna, Davide Marchese, Simone Bellavia, Aldobrando Broccolini, Riccardo Di Iorio, Giacomo Della Marca, Valerio Brunetti, Francesco Franceschi, Mauro Monforte, Paolo Calabresi and Giovanni Frisullo in European Stroke Journal

sj-docx-7-eso-10.1177_23969873251338654 – Supplemental material for A novel stroke mimic prediction score during in-hospital triage for suspected stroke patients: The Stroke Mimics Score (SMS) Supplemental material, sj-docx-7-eso-10.1177_23969873251338654 for A novel stroke mimic prediction score during in-hospital triage for suspected stroke patients: The Stroke Mimics Score (SMS) by Irene Scala, Marcello Covino, Pier Andrea Rizzo, Maurizio Bisegna, Davide Marchese, Simone Bellavia, Aldobrando Broccolini, Riccardo Di Iorio, Giacomo Della Marca, Valerio Brunetti, Francesco Franceschi, Mauro Monforte, Paolo Calabresi and Giovanni Frisullo in European Stroke Journal

sj-docx-8-eso-10.1177_23969873251338654 – Supplemental material for A novel stroke mimic prediction score during in-hospital triage for suspected stroke patients: The Stroke Mimics Score (SMS) Supplemental material, sj-docx-8-eso-10.1177_23969873251338654 for A novel stroke mimic prediction score during in-hospital triage for suspected stroke patients: The Stroke Mimics Score (SMS) by Irene Scala, Marcello Covino, Pier Andrea Rizzo, Maurizio Bisegna, Davide Marchese, Simone Bellavia, Aldobrando Broccolini, Riccardo Di Iorio, Giacomo Della Marca, Valerio Brunetti, Francesco Franceschi, Mauro Monforte, Paolo Calabresi and Giovanni Frisullo in European Stroke Journal

sj-docx-9-eso-10.1177_23969873251338654 – Supplemental material for A novel stroke mimic prediction score during in-hospital triage for suspected stroke patients: The Stroke Mimics Score (SMS) Supplemental material, sj-docx-9-eso-10.1177_23969873251338654 for A novel stroke mimic prediction score during in-hospital triage for suspected stroke patients: The Stroke Mimics Score (SMS) by Irene Scala, Marcello Covino, Pier Andrea Rizzo, Maurizio Bisegna, Davide Marchese, Simone Bellavia, Aldobrando Broccolini, Riccardo Di Iorio, Giacomo Della Marca, Valerio Brunetti, Francesco Franceschi, Mauro Monforte, Paolo Calabresi and Giovanni Frisullo in European Stroke Journal
