# Supplementary material for: A novel stroke mimic prediction score during in-hospital triage for suspected stroke patients: The Stroke Mimics Score (SMS)
Source: Eur Stroke J. 2025 May 15;10(4):1462–71. doi: 10.1177/23969873251338654 (PMC12084216; doi:10.1177/23969873251338654)
Supplement: sj-docx-1-eso-10.1177_23969873251338654 – Supplemental material for A novel stroke mimic prediction score during in-hospital triage for suspected stroke patients: The Stroke Mimics Score (SMS) [file sj-docx-1-eso-10.1177_23969873251338654.docx]

| **FABS** | |  | **TMS** | |
| --- | --- | --- | --- | --- |
| Absence of facial droop | +1 |  | Age | +0.2/year |
| Age <50 years | +1 |  | Atrial Fibrillation | +6 |
| Absence of atrial fibrillation | +1 |  | Hypertension | +3 |
| Systolic Blood pressure <150 mmHg | +1 |  | Seizure | -6 |
| Presence of isolated sensory deficit | +1 |  | Facial Weakness | +9 |
| History of seizure disorder | +1 |  | NIHSS>14 | +5 |

**Table S1.** Items and corresponding score points of the FABS and TMS scores. Abbreviations: FABS, absence of Facial droop, negative history of Atrial fibrillation, Age <50 years, systolic Blood pressure <150 mm Hg at presentation, history of Seizures, and isolated Sensory symptoms without weakness at presentation; TMS, TeleStrokeMimic; NIHSS, National Institutes of Health Stroke Scale.
