# Supplementary material for: A novel stroke mimic prediction score during in-hospital triage for suspected stroke patients: The Stroke Mimics Score (SMS)
Source: Eur Stroke J. 2025 May 15;10(4):1462–71. doi: 10.1177/23969873251338654 (PMC12084216; doi:10.1177/23969873251338654)
Supplement: sj-docx-2-eso-10.1177_23969873251338654 – Supplemental material for A novel stroke mimic prediction score during in-hospital triage for suspected stroke patients: The Stroke Mimics Score (SMS) [file sj-docx-2-eso-10.1177_23969873251338654.docx]

|  | | **Whole population**  (n = 8648) | **Derivation cohort**  (n = 6998) | **Validation Cohort**  (n = 1650) | **p-value** |
| --- | --- | --- | --- | --- | --- |
| **Demographics** | Age (years) | 75.0 (62.0-83.0) | 74.0 (61.0-83.0) | 78.0 (68.0-85.0) | **<0.001** |
|  | Sex (female) | 4278 (49.5%) | 3420 (48.9%) | 858 (52.0%) | **0.022** |
| **Triage** | Emergency | 3531 (40.8%) | 2802 (40.0%) | 729 (44.2%) | **0.001** |
|  | Urgency | 3926 (45.4%) | 3195 (45.7%) | 731 (44.3%) |  |
|  | Minor Urgency | 1191 (13.8%) | 1001 (14.3%) | 190 (11.5%) |  |
| **Mode of ED arrival** | Emergency Medical Service | 4649 (53.8%) | 3678 (52.6%) | 971 (58.8%) | **<0.001** |
| **Onset to door time** | <3 hours | 4376 (50.6%) | 3548 (50.7%) | 828 (50.2%) | 0.339 |
|  | 3-6 hours | 1583 (18.3%) | 1258 (18.0%) | 325 (19.7%) |  |
|  | 6-12 hours | 796 (9.2%) | 642 (9.2%) | 154 (9.3%) |  |
|  | 12-24 hours | 1893 (21.9%) | 1550 (22.2%) | 343 (20.8%) |  |
| **Vitals**  (ED admission) | Heart rate (bpm) | 81.0 (71.0-93.0) | 81.0 (71.0-92.0) | 81.0 (71.0-94.0) | 0.138 |
|  | Systolic blood pressure (mmHg) | 148.0 (130.0-166.0) | 152.5 (135.0-170.0) | 130.0 (110.0-140.0) | **<0.001** |
|  | Diastolic blood pressure (mmHg) | 84.0 (74.0-95.0) | 86.0 (77.0-97.0) | 75.0 (65.0-86.0) | **<0.001** |
|  | SaO2 (%) | 97.0 (95.0-98.0) | 97.0 (96.0-98.0) | 94.0 (92.0-95.0) | **<0.001** |
| **Neurological symptoms**  (ED admission) | NIHSS | 8.0 (3.0-16.0) | 8.0 (3.0-16.0) | 7.0 (3.0-16.0) | 0.762 |
|  | Altered consciousness | 1214 (14.0%) | 917 (13.1%) | 297 (18.0%) | **<0.001** |
|  | Confusional state | 1791 (20.7%) | 1409 (20.1%) | 382 (23.2%) | **0.007** |
|  | Language disorder | 4468 (51.7%) | 3645 (52.1%) | 823 (49.9%) | 0.106 |
|  | Motor impairment | 5007 (57.9%) | 4068 (58.1%) | 939 (56.9%) | 0.366 |
|  | Sensory impairment | 954 (11.0%) | 852 (12.2%) | 102 (6.2%) | **<0.001** |
|  | Isolated sensory impairment | 196 (2.3%) | 161 (2.3%) | 35 (2.1%) | 0.660 |
|  | Facial drop | 1187 (13.7%) | 933 (13.3%) | 254 (15.4%) | **0.029** |
|  | Headache | 1011 (11.7%) | 898 (12.8%) | 113 (6.8%) | **<0.001** |
|  | Seizure | 570 (6.6%) | 448 (6.4%) | 122 (7.4%) | 0.144 |
|  | Dizziness | 641 (7.4%) | 554 (7.9%) | 87 (5.3%) | **<0.001** |
|  | Syncope | 867 (10.0%) | 682 (9.7%) | 185 (11.2%) | *0.074* |
| **Comorbidities** | Charlson Comorbidity Index | 3.0 (2.0-5.0) | 3.0 (1.0-5.0) | 4.0 (2.0-6.0) | **<0.001** |
|  | History of CAD | 2250 (26.0%) | 1853 (26.5%) | 397 (24.1%) | **0.044** |
|  | Hypertension | 6030 (76.2%) | 4827 (76.0%) | 1203 (77.0%) | 0.401 |
|  | Atrial Fibrillation | 1044 (12.1%) | 834 (11.9%) | 210 (12.7%) | 0.364 |
|  | Congestive heart failure | 2517 (29.1%) | 2031 (29.0%) | 486 (29.5%) | 0.728 |
|  | Peripheral artery disease | 1417 (16.4%) | 1113 (15.9%) | 304 (18.4%) | **0.013** |
|  | Previous TIA/Stroke | 3195 (36.9%) | 2536 (36.2%) | 659 (39.9%) | **0.005** |
|  | Major neurocognitive disorder | 487 (5.6%) | 338 (4.8%) | 149 (9.0%) | **<0.001** |
|  | COPD | 342 (4.0%) | 228 (3.3%) | 114 (6.9%) | **<0.001** |
|  | Liver disease | 112 (1.3%) | 82 (1.2%) | 30 (1.8%) | **0.037** |
|  | Diabetes | 1370 (15.8%) | 1101 (15.7%) | 269 (16.3%) | 0.568 |
|  | Kidney failure | 2082 (24.1%) | 1723 (24.6%) | 359 (21.8%) | **0.014** |
|  | Active cancer | 548 (6.3%) | 421 (6.0%) | 127 (7.7%) | **0.012** |
| **Revascularization treatments** | Thrombolysis | 625 (7.2%) | 512 (7.3%) | 113 (6.8%) | 0.509 |
|  | Thrombectomy | 457 (5.3%) | 373 (5.3%) | 84 (5.1%) | 0.696 |
|  | Revascularization Treatments | 925 (10.7%) | 758 (10.8%) | 167 (10.1%) | 0.401 |
| **Neuroradiological examinations** | Brain MRI | 5206 (60.2%) | 4128 (59.0%) | 1078 (64.7%) | **<0.001** |
| **Outcomes** | Hospitalization | 6134 (70.9%) | 4894 (69.9%) | 1240 (75.2%) | **<0.001** |
|  | Hospitalization in Neurology department | 2915 (33.7%) | 2449 (35.0%) | 466 (28.2%) | **<0.001** |
|  | Hospitalization length (days) | 5.4 (0.8-10.6) | 5.2 (0.7-10.1) | 6.5 (1.1-13.4) | **<0.001** |
|  | In-hospital death | 763 (9.6%) | 554 (8.6%) | 209 (14.1%) | **<0.001** |
| **Scores for differential diagnosis** | FABS score | 2.0 (2.0-3.0) | 2.0 (2.0-3.0) | 3.0 (2.0-3.0) | **<0.001** |
|  | Telestroke mimic score | 15.0 (11.0-17.0) | 14.0 (11.0-17.0) | 16.0 (13.0-17.0) | **<0.001** |
|  | Stroke Mimic Score | 7.0 (6.0-9.0) | 7.0 (6.0-9.0) | 7.0 (6.0-9.0) | **<0.001** |
| **Discharge diagnosis** | Ischemic stroke | 3641 (42.1%) | 2921 (41.7%) | 720 (43.6%) | **<0.001** |
|  | TIA | 391 (4.5%) | 339 (4.8%) | 52 (3.2%) |  |
|  | Brain hemorrhage | 528 (6.1%) | 472 (6.7%) | 56 (3.4%) |  |
|  | Mimics | 4048 (47.2%) | 3266 (46.7%) | 818 (49.6%) |  |

**Table S2.** A summary of the characteristics of the entire study population, with a comparison between derivation and validation cohorts. *Abbreviations: ED, Emergency Department; peripheral oxygen saturation; NIHSS, National Institutes of Stroke Scale; CAD, Coronary Artery Disease; TIA, Transient Ischemic Attack; COPD, Chronic Obstructive Pulmonary Disease; MRI, Magnetic Resonance Imagin.*
