# Supplementary material for: A novel stroke mimic prediction score during in-hospital triage for suspected stroke patients: The Stroke Mimics Score (SMS)
Source: Eur Stroke J. 2025 May 15;10(4):1462–71. doi: 10.1177/23969873251338654 (PMC12084216; doi:10.1177/23969873251338654)
Supplement: sj-docx-3-eso-10.1177_23969873251338654 – Supplemental material for A novel stroke mimic prediction score during in-hospital triage for suspected stroke patients: The Stroke Mimics Score (SMS) [file sj-docx-3-eso-10.1177_23969873251338654.docx]

|  |  | **Derivation cohort**  (n = 6998) | **CVEs**  (n = 3732) | **Stroke Mimics**  (n = 3266) | **Unadjusted p value** |
| --- | --- | --- | --- | --- | --- |
| **Revascularization treatments** | Thrombolysis | 512 (7.3%) | 492 (13.2%) | 34 (1.0%) | **<0.001** |
|  | Thrombectomy | 373 (5.3%) | 351 (9.4%) | 0 (0.0%) | **<0.001** |
|  | Revascularization treatments | 758 (10.8%) | 724 (19.4%) | 34 (1.0%) | **<0.001** |
| **Neuroradiological examinations** | Brain MRI | 4128 (59.0%) | 2839 (76.1%) | 1289 (39.5%) | **<0.001** |
| **Outcomes** | Hospitalization | 4894 (69.9%) | 3358 (90.0%) | 1536 (47.0%) | **<0.001** |
|  | Hospitalization in the Neurology department | 2449 (35.0%) | 1929 (51.7%) | 520 (15.9%) | **<0.001** |
|  | Hospitalization length (days) | 5.2 (0.7-10.1) | 7.1 (4.0-12.3) | 1.0 (0.3-7.1) | **<0.001** |
|  | In-hospital death | 554 (8.6%) | 437 (12.9%) | 117 (3.9%) | **<0.001** |

**Table S3.** Comparison of revascularization treatments, neuroradiological examinations, and patient outcomes between patients in the derivation cohort diagnosed at discharge with CVEs and SMs. Abbreviations: CVEs, CerebroVascular Events; SMs, Stroke Mimics; MRI, Magnetic Resonance Imaging
