# Supplementary material for: A novel stroke mimic prediction score during in-hospital triage for suspected stroke patients: The Stroke Mimics Score (SMS)
Source: Eur Stroke J. 2025 May 15;10(4):1462–71. doi: 10.1177/23969873251338654 (PMC12084216; doi:10.1177/23969873251338654)
Supplement: sj-docx-4-eso-10.1177_23969873251338654 – Supplemental material for A novel stroke mimic prediction score during in-hospital triage for suspected stroke patients: The Stroke Mimics Score (SMS) [file sj-docx-4-eso-10.1177_23969873251338654.docx]

|  | | **Validation cohort**  (n = 1650) | **CVEs**  (n = 832) | **Mimics**  (n = 818) | **Unadjusted p value** |
| --- | --- | --- | --- | --- | --- |
| **Demographics** | Age (years) | 78.0 (68.0 – 85.0) | 80.0 (71.0 – 87.0) | 76.0 (65.0 – 83.0) | **<0.001** |
|  | Sex (female) | 792 (48.0%) | 395 (47.5%) | 397 (48.5%) | 0.667 |
| **Triage** | Emergency | 729 (44.2%) | 453 (54.4%) | 276 (33.7%) | **<0.001** |
|  | Urgency | 731 (44.3%) | 312 (37.5%) | 419 (51.2%) |  |
|  | Minor Urgency | 190 (11.5%) | 67 (8.1%) | 123 (15.0%) |  |
| **Mode of ED arrival** | Emergency Medical Service | 971 (58.8%) | 520 (62.5%) | 451 (55.1%) | **<0.001** |
| **Onset to door time** | <3 hours | 828 (50.2%) | 426 (51.2%) | 402 (49.1%) | 0.134 |
|  | 3-6 hours | 325 (19.7%) | 173 (20.8%) | 152 (18.6%) |  |
|  | 6-12 hours | 154 (9.3%) | 77 (9.3%) | 77 (9.4%) |  |
|  | 12-24 hours | 343 (20.8%) | 156 (18.7%) | 187 (22.9%) |  |
| **Vitals**  (ED admission) | Heart rate (bpm) | 81.0 (71.0 – 94.0) | 81.0 (71.0 - 93.0) | 82.0 (72.0 – 95.0) | 0.190 |
|  | Systolic blood pressure (mmHg) | 130.0 (110.0 – 140.0) | 130.0 (119.0 – 141.0) | 125.0 (105.8 – 140.0) | **<0.001** |
|  | Diastolic blood pressure (mmHg) | 75.0 (65.0 – 86.0) | 78.0 (67.0 – 87.0) | 73.0 (61.0 – 85.0) | **<0.001** |
|  | SaO2 (%) | 94.0 (92.0 – 95.0) | 94.0 (92.0 – 95.0) | 94.0 (92.0 – 95.0) | **0.005** |
| **Neurological symptoms**  (ED admission) | NIHSS | 7.0 (3.0 -16.0) | 7.0 (3.0 – 14.8) | 8.0 (3.0 – 17.0) | 0.297 |
|  | Altered consciousness | 297 (18.0%) | 165 (19.8%) | 132 (16.1%) | *0.051* |
|  | Confusional state | 382 (23.2%) | 151 (18.1%) | 231 (28.2%) | **<0.001** |
|  | Language disorder | 823 (49.9%) | 464 (55.8%) | 359 (43.9%) | **<0.001** |
|  | Motor impairment | 939 (56.9%) | 525 (63.1%) | 414 (50.6%) | **<0.001** |
|  | Sensory impairment | 102 (6.2%) | 30 (3.6%) | 72 (8.8%) | **<0.001** |
|  | Isolated sensory impairment | 35 (2.1%) | 10 (1.2%) | 25 (3.1%) | **0.009** |
|  | Facial drop | 254 (15.4%) | 138 (16.6%) | 116 (14.2%) | 0.176 |
|  | Headache | 113 (6.8%) | 33 (4.0%) | 80 (9.8%) | **<0.001** |
|  | Dizziness | 87 (5.3%) | 26 (3.1%) | 61 (7.5%) | **<0.001** |
|  | Seizure | 122 (7.4%) | 33 (4.0%) | 89 (10.9%) | **<0.001** |
|  | Syncope | 185 (11.2%) | 58 (7.0%) | 127 (15.5%) | **<0.001** |
| **Comorbidities** | Charlson Comorbidity Index | 4.0 (2.0 – 6.0) | 4.0 (2.0 – 6.0) | 3.0 (1.0 – 5.0) | **<0.001** |
|  | Previous Stroke/TIA | 659 (39.9%) | 499 (60.0%) | 160 (19.6%) | **<0.001** |
|  | History of CAD | 397 (24.1%) | 214 (25.7%) | 183 (22.4%) | 0.112 |
|  | Hypertension | 1203 (77.0%) | 650 (80.7%) | 553 (73.0%) | **<0.001** |
|  | Atrial Fibrillation | 210 (12.7%) | 103 (12.4%) | 729 (13.1%) | 0.699 |
|  | Heart failure | 486 (29.5%) | 210 (25.2%) | 276 (33.7%) | **<0.001** |
|  | Peripheral artery disease | 304 (18.4%) | 194 (23.3%) | 110 (13.4%) | **<0.001** |
|  | Major neurocognitive disorder | 149 (9.0%) | 59 (7.1%) | 90 (11.0%) | **0.006** |
|  | COPD | 114 (6.9%) | 53 (6.4%) | 61 (7.5%) | 0.384 |
|  | Connective tissue disease | 17 (1.0%) | 12 (1.4%) | 5 (0.6%) | *0.095* |
|  | Liver disease | 30 (1.8%) | 9 (1.1%) | 21 (2.6%) | **0.024** |
|  | Diabetes | 269 (16.3%) | 147 (17.7%) | 122 (14.9%) | 0.130 |
|  | Kidney failure | 359 (21.8%) | 137 (16.5%) | 222 (27.1%) | **<0.001** |
|  | Active cancer | 127 (7.7%) | 61 (7.3%) | 66 (8.1%) | 0.575 |
| **Revascularization treatments** | Thrombolysis | 113 (6.8%) | 108 (13.0%) | 8 (1.0%) | **<0.001** |
|  | Thrombectomy | 81 (4.9%) | 81 (9.7%) | 0 (0%) | **<0.001** |
|  | Thrombolysis and/or thrombectomy | 167 (10.1%) | 159 (19.1%) | 8 (1.0%) | **<0.001** |
| **Neuroradiological examinations** | Brain MRI | 1078 (64.7%) | 660 (79.3%) | 418 (51.1%) | **<0.001** |
| **Outcomes** | Hospitalization | 1240 (75.2%) | 761 (91.5%) | 479 (58.6%) | **<0.001** |
|  | Hospitalization in Neurology department | 466 (28.2%) | 367 (44.1%) | 99 (12.1%) | **<0.001** |
|  | Hospitalization length (days) | 6.5 (1.1 – 13.4) | 8.3 (4.5 – 16.4) | 3.3 (0.4 – 10.6) | **<0.001** |
|  | Need of mechanical ventilation | 70 (5.7%) | 46 (8.,0%) | 24 (3.7%) | **0.001** |
|  | In-hospital death | 209 (14.1%) | 145 (19.7%) | 64 (8.6%) | **<0.001** |
| **Scores for stroke diagnosis (vs stroke mimics)** | FABS score | 3.0 (2.0 – 3.0) | 3.0 (2.0 – 3.0) | 3.0 (2.0 – 3.0) | **<0.001** |
|  | TSM score | 16.0 (13.0 – 17.0) | 16.0 (14.0 – 18.0) | 15.0 (12.0 – 17.0) | **<0.001** |
|  | SMS | 7.0 (6.0 – 9.0) | 8.0 (7.0 – 9.0) | 6.0 (5.0 – 7.0) | **<0.001** |

**Table S4.** A comparison of demographics, clinical variables, outcomes, and median scores for distinguishing CVEs from SM in the validation cohort. Abbreviations: CVEs, Cerebrovascular Events; ED, Emergency Department; bpm, beats per minute; SaO2, Peripheral oxygen saturation; NIHSS, National Institutes of Stroke Scale; CAD, Coronary Artery Disease; TIA, Transient Ischemic Attack; COPD, Chronic Obstructive Pulmonary Disease; MRI, Magnetic Resonance Imaging; TMS, TeleStroke Mimic Score; SMS, Stroke Mimic Score; IQR, InterQuartile Range.
