# Supplementary material for: A novel stroke mimic prediction score during in-hospital triage for suspected stroke patients: The Stroke Mimics Score (SMS)
Source: Eur Stroke J. 2025 May 15;10(4):1462–71. doi: 10.1177/23969873251338654 (PMC12084216; doi:10.1177/23969873251338654)
Supplement: sj-docx-5-eso-10.1177_23969873251338654 – Supplemental material for A novel stroke mimic prediction score during in-hospital triage for suspected stroke patients: The Stroke Mimics Score (SMS) [file sj-docx-5-eso-10.1177_23969873251338654.docx]

|  | **Unstandardized**  **Coefficients** | | **Standardized**  **Coefficients** | |  |
| --- | --- | --- | --- | --- | --- |
|  | **β** | **Standard error** | **β** | **P value** | **Item value assigned** |
| Age >71 years | 0.111 | 0.011 | 0.110 | <0.001 | +1 |
| SBP ≥140 mmHg at onset | 0.050 | 0.011 | 0.049 | <0.001 | +1 |
| No seizure at onset | 0.250 | 0.021 | 0.122 | <0.001 | +1 |
| No confusional state at onset | 0.125 | 0013 | 0.100 | <0.001 | +1 |
| No syncope at onset | 0.145 | 0.018 | 0.086 | <0.001 | +1 |
| No isolated sensory disorders | 0.156 | 0.016 | 0.102 | <0.001 | +1 |
| Motor disorders at onset | 0.077 | 0.011 | 0.076 | <0.001 | +1 |
| No headache at onset | 0.117 | 0.016 | 0.079 | <0.001 | +1 |
| Clinical history of stroke/TIA | 0.371 | 0.011 | 0.358 | <0.001 | +3 |
| Clinical history of CAD | 0.061 | 0.012 | 0.054 | <0.001 | +1 |

**Table S5.** Linear regression model to assign score points to each independent predictor of cerebrovascular events at discharge. Abbreviations: SBP, Systolic Blood Pressure; TIA, Transient Ischemic Attack; CAD, Coronary Artery Disease
