# Supplementary material for: A novel stroke mimic prediction score during in-hospital triage for suspected stroke patients: The Stroke Mimics Score (SMS)
Source: Eur Stroke J. 2025 May 15;10(4):1462–71. doi: 10.1177/23969873251338654 (PMC12084216; doi:10.1177/23969873251338654)
Supplement: sj-docx-6-eso-10.1177_23969873251338654 – Supplemental material for A novel stroke mimic prediction score during in-hospital triage for suspected stroke patients: The Stroke Mimics Score (SMS) [file sj-docx-6-eso-10.1177_23969873251338654.docx]

| **Score Value** | **Stroke Mimic Score** | | **FABS** | | **TeleStroke Mimic Score** | |
| --- | --- | --- | --- | --- | --- | --- |
| 0 | Low-risk | 0/0 (0.0%) | High-risk | 59/76 (77.6%) | Low-risk | 0/3 (0.0%) |
| 1 |  | 0/2 (0.0%) |  | 637/913 (69.8%) |  | 0/4 (0.0%) |
| 2 |  | 1/10 (10.0%) | Moderate-risk | 1968/3284 (59.9%) |  | 1/7 (14.3%) |
| 3 |  | 7/105 (6.7%) |  | 935/2057 (45.5%) |  | 4/14 (28.6%) |
| 4 |  | 43/406 (10.6%) | Low-risk | 129/598 (21.6%) |  | 4/34 (11.8%) |
| 5 |  | 201/854 (23.5%) |  | 4/70 (5.7%) |  | 3/40 (7.5%) |
| 6 | Moderate-risk | 449/1226 (36.6%) |  | |  | 10/69 (14.5%) |
| 7 |  | 599/1212 (49.4 %) |  |  |  | 17/130 (13.1%) |
| 8 |  | 576/911 (63.2%) |  |  | Moderate-risk | 60/266 (22.6%) |
| 9 | High-risk | 611/808 (75.6%) |  |  |  | 94/371 (25.3%) |
| 10 |  | 735/889 (82.7%) |  |  |  | 175/494 (35.4%) |
| 11 |  | 445/501 (88.8%) |  |  |  | 209/542 (38.6%) |
| 12 |  | 65/74 (87.8%) |  |  | High-risk | 239/502 (47.6%) |
| 13 |  | |  |  |  | 256/502 (51.0%) |
| 14 |  |  |  |  |  | 304/555 (54.8%) |
| 15 |  |  |  |  |  | 507/813 (62.4%) |
| 16 |  |  |  |  |  | 596/899 (66.3%) |
| 17 |  |  |  |  |  | 508/723 (70.3%) |
| 18 |  |  |  |  |  | 301/408 (73.8%) |
| 19 |  |  |  |  |  | 89/125 (71.2%) |
| 20 |  |  |  |  |  | 33/49 (67.3%) |
| 21 |  |  |  |  |  | 31/48 (64.6%) |
| 22 |  |  |  |  |  | 42/58 (72.4%) |
| 23 |  |  |  |  |  | 44/67 (65.7%) |
| 24 |  |  |  |  |  | 71/100 (71.0%) |
| 25 |  |  |  |  |  | 60/80 (75.0%) |
| 26 |  |  |  |  |  | 39/53 (73.6%) |
| 27 |  |  |  |  |  | 24/30 (80.0%) |
| 28 |  |  |  |  |  | 10/11 (90.9%) |
| 29 |  |  |  |  |  | 1/1 (100.0%) |

**Tabel S6.** Number of CVE diagnoses over the total number of patients with a given SMS score value in the retrospective derivation cohort (first column). The second and third columns show the same analyses for FABS and TMS scores.. Based on the probability of a discharge diagnosis of CVEs, the scores were divided into three risk categories (i.e. low, moderate, and high risk). Abbreviations: CVEs, Cerebrovascular Events
