# Supplementary material for: A novel stroke mimic prediction score during in-hospital triage for suspected stroke patients: The Stroke Mimics Score (SMS)
Source: Eur Stroke J. 2025 May 15;10(4):1462–71. doi: 10.1177/23969873251338654 (PMC12084216; doi:10.1177/23969873251338654)
Supplement: sj-docx-7-eso-10.1177_23969873251338654 – Supplemental material for A novel stroke mimic prediction score during in-hospital triage for suspected stroke patients: The Stroke Mimics Score (SMS) [file sj-docx-7-eso-10.1177_23969873251338654.docx]

| **Score Value** | **SMS** | | **FABS** | | **TSM** | |
| --- | --- | --- | --- | --- | --- | --- |
| 0 | Low stroke risk | 0/0 | High stroke risk | 8/9 (88.9%) | Low stroke risk | 0/1 (0.0%) |
| 1 |  | 0/0 |  | 58/105 (55.2%) |  | 0/0 |
| 2 |  | 1/5 (20.0%) | Medium stroke risk | 326/549 (59.4%) |  | 0/1 (0.0%) |
| 3 |  | 3/20 (15.0%) |  | 403/825 (48.8%) |  | 0/2 (0.0%) |
| 4 |  | 7/88 (8.0%) | Low stroke risk | 37/147 (25.2%) |  | 0/3 (0.0%) |
| 5 |  | 51/244 (20.9%) |  | 0/15 (0.0%) |  | 3/8 (37.5%) |
| 6 | Medium stroke risk | 120/341 (35.2%) |  | |  | 2/6 (33.3%) |
| 7 |  | 125/262 (47.7%) |  |  |  | 3/8 (37.5%) |
| 8 |  | 113/177 (63.8%) |  |  | Medium stroke risk | 3/15 (20.0%) |
| 9 | High stroke risk | 208/266 (78.2%) |  |  |  | 11/47 (23.4%) |
| 10 |  | 180/214 (84.1%) |  |  |  | 10/58 (17.2%) |
| 11 |  | 24/33 (72.7%) |  |  |  | 25/90 (27.8%) |
| 12 |  | 0/0 |  |  | High stroke risk | 55/118 (46.6%) |
| 13 |  | |  |  |  | 57/130 (43.8%) |
| 14 |  |  |  |  |  | 60/125 (48.0%) |
| 15 |  |  |  |  |  | 100/209 (47.8%) |
| 16 |  |  |  |  |  | 105/220 (47.7%) |
| 17 |  |  |  |  |  | 142/239 (59.4%) |
| 18 |  |  |  |  |  | 104/144 (72.2%) |
| 19 |  |  |  |  |  | 39/53 (73.6%) |
| 20 |  |  |  |  |  | 24/25 (96.0%) |
| 21 |  |  |  |  |  | 15/20 (75.0%) |
| 22 |  |  |  |  |  | 11/18 (61.1%) |
| 23 |  |  |  |  |  | 9/17 (52.9%) |
| 24 |  |  |  |  |  | 13/24 (54.2%) |
| 25 |  |  |  |  |  | 21/35 (60.0%) |
| 26 |  |  |  |  |  | 16/26 (61.5%) |
| 27 |  |  |  |  |  | 3/7 (42.9%) |
| 28 |  |  |  |  |  | 1/1 (100.0%) |
| 29 |  |  |  |  |  | 0/0 |

**Table S7.** Number of CVE diagnoses over the total number of patients with a given SMS score value in the validation cohort(first column). The second and third columns present the same analysis for the FABS and TMS scores.. Based on the obtained probability of a discharge diagnosis of CVEs, the three scores were divided into three risk categories (low, moderate, and high risk). *Abbreviations: CVEs, Cerebrovascular Events; SMS, Stroke Mimics Score; TMS, TelestrokeMimic Score*
