# Supplementary material for: A novel stroke mimic prediction score during in-hospital triage for suspected stroke patients: The Stroke Mimics Score (SMS)
Source: Eur Stroke J. 2025 May 15;10(4):1462–71. doi: 10.1177/23969873251338654 (PMC12084216; doi:10.1177/23969873251338654)
Supplement: sj-docx-8-eso-10.1177_23969873251338654 – Supplemental material for A novel stroke mimic prediction score during in-hospital triage for suspected stroke patients: The Stroke Mimics Score (SMS) [file sj-docx-8-eso-10.1177_23969873251338654.docx]

| **Score** | **AUROC (95% CI)** | **p** |
| --- | --- | --- |
| SMS | 0.774 (0.752-0.797) | **<0.001** |
| SMSg | 0.744 (0.721-0.768) | **<0.001** |
| FABS | 0.595 (0.568-0.628) | **<0.001** |
| FABSg | 0.560 (0.532-0.587) | **<0.001** |
| TMS | 0.649 (0.622-0.675) | **<0.001** |
| TMSg | 0.577 (0.549-0.604) | **<0.001** |

**Table S8.** Diagnostic accuracy of all tested scores in the prospective validation cohort calculated using ROC curves. *Abbreviations: AUROC, Area Under the Receiver Operating Characteristic; CI, Confidence Interval; SMS, Stroke Mimic Score; SMS, Stroke Mimic Score grouped version; FABSg, FABS grouped version; TMS, Telestroke Mimic Score; TMSg, Telestroke Mimic Score grouped version.*
