# Supplementary material for: A novel stroke mimic prediction score during in-hospital triage for suspected stroke patients: The Stroke Mimics Score (SMS)
Source: Eur Stroke J. 2025 May 15;10(4):1462–71. doi: 10.1177/23969873251338654 (PMC12084216; doi:10.1177/23969873251338654)
Supplement: sj-docx-9-eso-10.1177_23969873251338654 – Supplemental material for A novel stroke mimic prediction score during in-hospital triage for suspected stroke patients: The Stroke Mimics Score (SMS) [file sj-docx-9-eso-10.1177_23969873251338654.docx]

| **Pairwise comparison of ROC curves** | **Difference between AOUROCs** | **Z-statistic** | **p-value** |
| --- | --- | --- | --- |
| SMS vs FABS | 0.144 (CI 0.132-0.156) | 23.707 | <0.001 |
| SMSg vs FABSg | 0.151 (0.140-0.161) | 27.317 | <0.001 |
| SMS vs TMS | 0.0955 (CI 0.084-0.107) | 16.130 | <0.001 |
| SMSg vs TMSg | 0.116 (0.105-0.126) | 21.474 | <0.001 |
| FABS vs TMS | 0.0487 (0.036-0.062) | 7.430 | <0.001 |
| FABSg vs TMSg | 0.0347 (0.024-0.045) | 6.563 | <0.001 |

**Table S9.** Comparison of the Area Under the Curve of the ROC curves for the continuous and grouped versions of the scores in the retrospective cohort of patients (derivation cohort) using the DeLong method. Abbreviations: ROC, Receiver-Operating Characteristic; AUROC, Area Under the ROC curves; SMS, Stroke Mimic Score; SMSg, Stroke Mimic Score grouped version; FABSg, FABS score grouped version; TMS, Telestroke Mimic Score; TMS, Telestroke Mimic Score grouped version.
